# Co-Formulation of Recombinant Porcine IL-18 Enhances the Onset of Immune Response in a New *Lawsonia intracellularis* Vaccine

**DOI:** 10.3390/vaccines11121788

**Published:** 2023-11-30

**Authors:** Angela Hidalgo-Gajardo, Nicolás Gutiérrez, Emilio Lamazares, Felipe Espinoza, Fernanda Escobar-Riquelme, María J. Leiva, Carla Villavicencio, Karel Mena-Ulecia, Raquel Montesino, Claudia Altamirano, Oliberto Sánchez, Coralia I. Rivas, Álvaro Ruíz, Jorge R. Toledo

**Affiliations:** 1 Laboratorio de Biotecnología y Biofármacos, Departamento de Fisiopatología, Facultad de Ciencias Biológicas, Universidad de Concepción, VIII Región, Concepción 4070386, Chile; angehidalgo@udec.cl (A.H.-G.); mariajleiva@udec.cl (M.J.L.); cvillavicen2019@udec.cl (C.V.); corivas@udec.cl (C.I.R.); 2 Centro de Desarrollo e Innovación Biovacuvet SpA, VIII Región, Concepción 4090838, Chile; 3 Departamento de Ciencias Biológicas y Químicas, Facultad de Recursos Naturales, Universidad Católica de Temuco, IX Región, Temuco 4813302, Chile; kmena@uct.cl; 4 Laboratorio de Cultivos Celulares, Escuela de Ingeniería Bioquímica, Pontificia Universidad Católica de Valparaíso, V Región, Valparaíso 2362803, Chile; claudia.altamirano@pucv.cl; 5 Departamento de Patología y Medicina Preventiva, Facultad de Ciencias Veterinarias, Universidad de Concepción, XVI Región, Chillán 3812120, Chile; aruiz@udec.cl

**Keywords:** cytokine, interleukin 18 (IL-18), adjuvant, *Lawsonia intracellularis*, *Pichia pastoris*, porcine enteropathy

## Abstract

Pig is one of the most consumed meats worldwide. One of the main conditions for pig production is Porcine Enteropathy caused by *Lawsonia intracellularis*. Among the effects of this disease is chronic mild diarrhea, which affects the weight gain of pigs, generating economic losses. Vaccines available to prevent this condition do not have the desired effect, but this limitation can be overcome using adjuvants. Pro-inflammatory cytokines, such as interleukin 18 (IL-18), can improve an immune response, reducing the immune window of protection. In this study, recombinant porcine IL-18 was produced and expressed in *Escherichia coli* and *Pichia pastoris*. The protein’s biological activity was assessed in vitro and in vivo, and we determined that the *P. pastoris* protein had better immunostimulatory activity. A vaccine candidate against *L. intracellularis,* formulated with and without IL-18, was used to determine the pigs’ cellular and humoral immune responses. Animals injected with the candidate vaccine co-formulated with IL-18 showed a significant increase of Th1 immune response markers and an earlier increase of antibodies than those vaccinated without the cytokine. This suggests that IL-18 acts as an immunostimulant and vaccine adjuvant to boost the immune response against the antigens, reducing the therapeutic window of recombinant protein-based vaccines.

## 1. Introduction

According to the Food and Agriculture Organization of the United Nations, pig is the second most consumed meat (34%), followed by beef and sheep meat. Pork meat production is expected to rise to 127 Mt by 2030, up 13% from a base level in 2018–2020 [1]. These data are relevant to the health and disease control of facilities holding a porcine population [2,3].

Porcine Enteropathy (PE) is one of the most economically significant diseases in facilities of porcine production and is caused by the obligate intracellular bacteria *Lawsonia intracellularis* [4,5,6]. This bacterium is a primary invader, preferring to grow in epithelial cells’ cytoplasm from the pig intestinal tract. The two most important clinical manifestations of PE are hemorrhagic diarrhea, which causes the sudden death of animals, and mild chronic diarrhea, which delays the growth of fattening pigs [7,8,9].

There are vaccines against *L. intracellularis.* These consist of live attenuated bacteria grown in cell cultures and are orally administered [3,10]. However, administering this vaccine requires animals to be without a pulse of antibiotics for about a week before application. This incurs the risk of a reversion to virulence [11], a significant disadvantage of attenuated vaccines. However, while subunit vaccines do not have this problem, they do not stimulate the immune system properly, mainly due to the low activation of B-lymphocytes and antigen-presenting cells [12,13]. The use of adjuvants that enhance the immune-stimulatory activity of vaccines is one strategy by which to improve this disadvantage [14].

Adjuvants enhance a specific immune response’s magnitude, quality, and durability, ideally with minimal toxicity [15,16]. Adding adjuvants might reduce the antigen dose and the number of immunizations, improve the antigen’s immunogenicity, and positively modulate the immune response [17,18].

Cytokines are an attractive molecular adjuvant alternative for subunit vaccines, which are molecules that naturally modulate the immune system responsible for intercellular communication [19]. These molecules could trigger both the innate and adaptive immune responses, which include antigen-presenting cells maturation; CD4+ cells differentiation, T helper 1 (Th1) and T helper 2 (Th2); and CD8+ and NK cells induction [20].

Interleukin 18 (IL-18) is a pro-inflammatory cytokine synthesized as an inactive precursor and is present in blood monocytes from healthy subjects. Additionally, IL-18 is present in the gastrointestinal tract’s epithelial cells, peritoneal macrophages, spleen, keratinocytes, and nearly all epithelial cells [21]. This precursor is processed intracellularly by caspase-1 into its mature, biologically active form of 18 kDa [22]. IL-18 is involved in the activation and differentiation of several T cell populations and enhances T and NK cell maturation, cytokine production, and Fas-L-mediated cytotoxicity, taking part in the cellular immune response to intracellular pathogens [23].

A multi-antigenic subunit vaccine candidate against *L. intracellularis* has been shown to stimulate antibody production and activate the cellular immune response of vaccinated pigs, protecting them from the intestinal damage caused by the bacteria. However, antibody production occurs at week four post-immunization (1 week after the booster), even using interferon alpha as an adjuvant [24].

Here, we produced a porcine recombinant IL-18, expressed in *Pichia pastoris* yeast, which can be used as a molecular adjuvant for recombinant vaccines to improve the humoral immune response in pigs, reducing the immunological window protection.

## 2. Materials and Methods

### 2.1. Porcine IL-18 Modeling by Homology

For understanding porcine IL-18 structural characteristics, a three-dimensional (3D) structure is essential. Unfortunately, the porcine IL-18 crystal structure has not been resolved yet. Therefore, we performed a comparative structure prediction approach to generate the 3D coordinates of the porcine IL-18 structure. According to the general criteria for this type of study [25,26,27,28], sequential stages were established and are described below.

A total of 157 amino acids of the porcine IL-18 sequence were used. The sequence was transformed into FASTA format through the CLUSTALX version 2.1 program [29]. The first homology modeling stage is the search for templates. The pig IL-18 amino acid sequence obtained in FASTA format was uploaded to the Swiss-Model server [30]. This web server is designed to quickly build and evaluate the protein homology models [30,31] Swiss-Model web server with the pig IL-18 sequence performed a BLAST version 2.13.0 [32,33] search followed by HHBLITS version 3.3.0 [34] to obtain all of the retrieved templates. The best templates to generate the models were selected, considering the identity percentage (above 70% identity) of the known proteins within the Protein Data Bank [35,36] and the best possible coverage.

With the selected template, models by homology were generated, and the Swiss-Model web server built the pig IL-18 3D model based on and validated by MolProbity [37] and QMEAN [38]. This parameter suggests whether the predicted IL-18 structure through the homology modeling approach is comparable to the expected structure using other experimental structures of similar size and structure [30,38]. To select the best models, we took the QMEAN as a reference value (0 to −4 range), where a QMEAN value around 0 suggests the high quality of the homology-modeled protein structure [38,39]. The structural analysis of the generated models and the figures were obtained using Pymol Version 2.3.0 [40,41].

### 2.2. E. coli Rupture and pIL-18 Expression

The mature porcine IL-18 nucleotide sequence (NCBI: NM_213997.1) was designed and linked to 6 histidine residues (6His-tag) at the N-terminal and cloned into the pET22b (+) vector (GenScript, Hong Kong, China). Protein expression was induced in a 5 L culture of *E. coli* BL-21 (DE3) strain transformed with the construct pET22b-pIL-18, with culture conditions of 37 °C, pH 7.0, and 0.5 mM isopropyl β-D1-thiogalactopyranoside (IPTG), for 5 h. Cells were harvested at 4260× *g* for 10 min at 4 °C. Cells were resuspended in NaH_2_PO_4_ buffer (50 mM), NaCl (300 mM), imidazole (3 mM), and urea (8 M). The solution was lysed by mechanical disruption in a high-pressure homogenizer (EmulsiFlex C-5, Avestin, Inc., Ottawa, ON, Canada). The sample was centrifuged at 17,500× *g* for 10 min at 4 °C for the soluble fraction recovery. A negative expression control culture was performed in the same conditions, using an empty pET22b (+) vector. The protein expressed in this system will be called epIL-18.

### 2.3. Cloning of pIL-18 in pPS10

The sequence of the mature IL-18 gene was obtained from the pUC57-pIL-18, previously cloned by GenScript (Hong Kong, China), using NaeI and ApaI restriction enzymes. Furthermore, it was cloned in pPS10 expression vector (from Dr. Oliberto Sánchez, Department of Pharmacology, Universidad de Concepción). The IL-18 gene presence was confirmed by restriction assay using the same restriction enzymes and visualizing a 515 bp fragment in an agarose gel.

### 2.4. pIL-18 Yeast Expression

The *P. pastoris* yeast was transformed by electroporation with the linearized pPS10-pIL-18 vector containing the methanol-inducible promoter of alcohol oxidase 1 (AOX1). The producer clone was identified by DNA Southern blot. The protein expressed in this system will be called ppIL-18. ppIL-18 protein expression was determined by Western blot after performing a 5 L fermentation culture at 30 °C, pH 4.5, and 800 rpm of agitation for the first phase. Twenty-four hours later, protein expression was induced with methanol. The induction phase was performed for 72 h, measuring biomass in dry weight (g/L) every 12 h. The culture was centrifuged at 6190× *g* for 10 min and the supernatant was concentrated 25 times using a 10 kDa filter. ppIL-18 was not purified because *P. pastoris* is a generally recognized as safe (GRAS) organism. The concentrated medium was quantified by densitometry in Western blot assay using a calibration curve with commercial pIL-18 (R&D Systems, Minneapolis, MN, USA).

### 2.5. SDS-PAGE and Western Blot

SDS-polyacrylamide gel electrophoresis (SDS-PAGE) analysis was performed in 15% gels and stained with Coomassie blue solution. For Western blot analysis, proteins were transferred onto a nitrocellulose membrane (Schleicher and Schuell, Dassel, Germany) using a semi-dry TransBlot-Turbo electro-blotter (Bio-Rad, Hercules, CA, USA). Mouse anti-pIL-18 monoclonal antibody (eBioscience, San Diego, CA, USA) was used as the primary antibody, and donkey anti-IgG mouse IRDye 800cw (LI-COR, Lincoln, NE, USA) was used as a secondary antibody. Infrared signals were measured using the Odyssey System from LI-COR Biosciences (Lincoln, NE, USA).

### 2.6. epIL-18 Purification by Immobilized Metal Affinity Chromatography (IMAC)

Soluble epIL-18 was purified by immobilized metal affinity chromatography (IMAC). The soluble fraction from *E. coli* rupture was loaded into a Ni^2+^ charged matrix IMAC SepharoseTM 6 Fast Flow (GE Healthcare, Chicago, IL, USA), connected to the GE ÄKTA prime plus system. The decreasing urea gradient (8 to 0 M) was completed to recover not-denaturing conditions. Elution was performed with increasing imidazole concentration (25, 250, and 500 mM). Purified epIL-18 was quantified by SDS-PAGE using bovine serum albumin as an external standard and the recovery percentage of the initial fraction was calculated by densitometry. Images were captured and processed using an LI-COR imaging system program (Image Studio v 3.1, LI-COR, Lincoln, NE, USA).

### 2.7. Pig Peripheral Blood Lymphocyte Extraction

Peripheral blood samples were taken from three-months-old healthy pigs for lymphocyte isolation using lymphocyte separation medium (LMS, Corning, Corning, NY, USA) following the manufacturer’s instructions. Lymphocytes were cultured in RPMI culture medium (Gibco, Thermo Scientific, Waltham, MA, USA) supplemented with 10% fetal bovine serum (Biological Industries, Beit-Haemek, Israel at 37 °C and 5% CO_2_ culture conditions.

### 2.8. Lymphocyte Proliferation Assay

Lymphocyte proliferation was measured using 3-(4.5-Dimetil-2-tiazolil)-2.5-diphenyl-2H-tetrazolio bromide (MTT) (Sigma Aldrich, Burlington, MA, USA). The cell treatment was performed in 96-well plates for 24 h, with different concentrations of each of the porcine recombinants IL-18, including controls without treatment and with the culture medium of non-transformed yeast. The viability assay was made by adding 1 mg/mL of MTT reagent in PBS to the culture medium and incubating for 1 h at 37 °C. Later, the formazan crystals were dissolved with isopropanol 100% at 37 °C for 15 min. Absorbance was measured at 560 nm in the SPECTROstar Nano (BMG Labtech, Ortenberg, Germany) multi-plate reader. Results were plotted in percentages concerning the control without treatment, corresponding to 100%.

### 2.9. Pro-Inflammatory Cytokines Analysis In Vitro by Real-Time PCR

Porcine peripheral blood mononuclear cells (PBMCs) were treated with each of the recombinant pIL-18 for 24 h. Total RNA was extracted using TRIzol (Ambion^®^, Life Technologies, Carlsbad, CA, USA) according to the manufacturer’s instructions. The PCR was performed using the commercial kit Brilliant II SYBR ^®^ Green qRT-PCR Master Mix (Agilent Technologies, Santa Clara, CA, USA) and the equipment Aria Mx (Agilent Technologies, Santa Clara, CA, USA). The comparative threshold cycle values were normalized for the β-actin reference gene, the group without treatment was used as the calibrator and the results were expressed as Ct relative quantification by 2^−ΔΔCT^ method [42].

### 2.10. Pro-Inflammatory Cytokines Analysis In Vivo by Real-Time PCR

epIL-18 and ppIL-18 biologic activity was tested in adult pigs (Duroc/Yorkshire). Three-month-old healthy piglets were separated randomly into 2 groups of 5 animals, and they were intramuscularly administered with 100 µg of epIL-18 or ppIL-18 emulsified in Montanide ISA 12 AVG (Seppic, Lons, France) in a ratio of 80:20 (water:oil). PBMCs were isolated from pig’s blood at days 0, 5, 10, 15, and 20 post-administration. The TRIzol reagent (Ambion^®^, Life Technologies, Carlsbad, CA, USA) method was used to do the RNA extraction. The qPCR assay was completed by the Agilent Brilliant II SYBR Green qRT-PCR kit (Agilent Technologies, Santa Clara, CA, USA). The pro-inflammatory cytokines Interferon gamma (IFN-γ), granulocyte macrophage colony-stimulating factor (GM-CSF), and tumor necrosis factor alpha (TNF-α), were measured using β-actin as a normalizer and day 0 of each pig as calibrator. The reactions were performed in the Aria Mx equipment (Agilent Technologies, Santa Clara, CA, USA), and the results are expressed as Ct relative quantification by 2^−ΔΔCT^ method [42].

### 2.11. Immunization Assay in Pigs

The immune response of ppIL-18 with or without *Lawsonia intracellularis* antigens [24] was evaluated in three-week-old healthy Duroc/Yorkshire piglets. ppIL-18 and the antigens for *L. intracellularis* were emulsified in Montanide ISA 50 v2 (Seppic, Lons, France) with a ratio of 60:40 (water:oil). The dose of 200 μg for antigens and 25 μg of pIL-18 per 1 mL of total volume was intramuscularly administered in the backside of the neck, using a 21G 1 ½ inch syringe. Four experimental groups of pigs were randomly gathered: (i) control (PBS buffer, *n* = 6), (ii) ppIL-18 (*n * = 6), (iii) *L. intracellularis* antigens (*n* = 8), and (iv) ppIL-18 co-formulated with *L. intracellularis* antigens (*n * = 8). The immunization scheme comprised one dose of the antigens on day 0 and a booster on day 20. ppIL-18 was only administered at day 0. Blood samples were collected on days 5, 10, 20, 30 and 40 after the immunization for ELISA and qRT-PCR assays.

### 2.12. Detection of Cellular Immune Response in Pigs

RNA isolated from peripheral blood lymphocytes was extracted using TRIzol (Ambion^®^, Life Technologies, Carlsbad, CA, USA) reagent. The mRNA relative expression levels for IL-12, IFN-γ, TNF-α and GM-CSF genes were measured by qRT-PCR using the Brilliant II SYBR Green qRT-PCR Master Mix (Agilent Technologies, Santa Clara, CA, USA). β-actin was used as a normalizer, and day 0 as a calibrator. Each relative expression was compared with the control (Day 0).

### 2.13. Humoral Immune Response Evaluation

Flat-bottom 96-well ELISA plates (Nunc, Thermo Fisher Scientific, Waltham, MA, USA) were coated with 100 ng per well of the antigens from *L. intracellularis.* Plates were washed with PBS 0.05% Tween 20 (PBST) and blocked with 3% skimmed milk in PBS. After washing, diluted serum from the pigs was added (100 μL/well) and incubated for 2 h at 37 °C. Plates were washed, and the 1/10,000 diluted goat anti-pig IgG-HRP polyclonal antibody (Abcam, Boston, MA, USA) was added. Plates were washed and revealed with a substrate solution of o-phenylenediamine dihydrochloride (OPD) 0.4 mg/mL (Sigma, Burlington, MA, USA). The absorbance at 450 nm was read using the Synergy™ HTX Multi-Mode Microplate Reader (BioTek, Winooski, VT, USA). Titer was determined using pre-immune serum absorbance value as cut-off multiplied by two, using serum sample serial dilutions (1:500 to 1:64,000), assigning titer value as indicative of the last dilution in which the antibody was detected.

### 2.14. Statistical Analysis

Statistical analysis was performed using GraphPad Prism Software version 8.0 (GraphPad Software, Boston, MA, USA). Depending on the data, the results were analyzed with one-way ANOVA or two-way ANOVA compared with the respective control. Significance was considered for *p* < 0.05.

## 3. Results

### 3.1. pIL-18 Modeling

**Figure 1:**
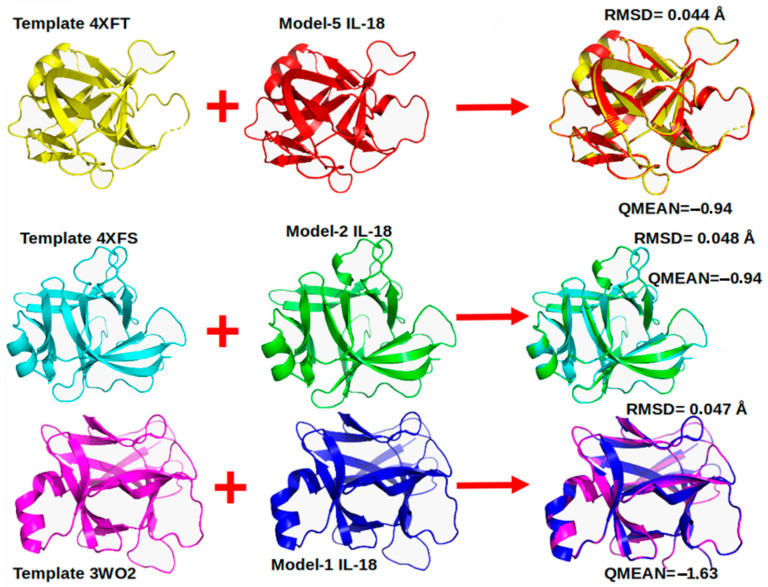
Graphic representation of the three best models obtained from the Swiss-Model with their respective templates.

Based on the amino acid sequence of the pig interleukin-18 using our analysis, we searched for the most suitable templates for the model’s construction by homology. We considered 57 templates through the Swiss-Model website. Eleven of the nineteen templates had an identity percentage higher than 72% concerning our IL-18 sequence (Table S1). These templates were selected for model generation to build the pig IL-18 3D structure. From these 11 templates, the 3D models were generated to construct the pig IL-18 structure (Figure 1).

### 3.2. Porcine IL-18 Expression in E. coli and P. pastoris

**Figure 2:**
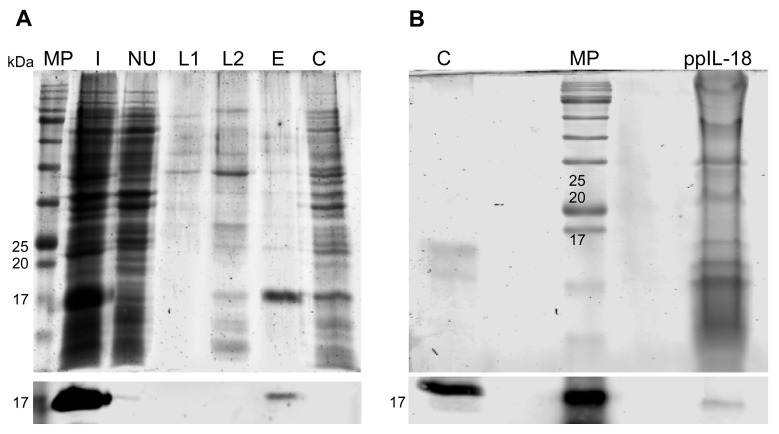
Recombinant porcine IL-18 production. (**A**) Purification and renaturation of the pIL-18 expressed in *E. coli*. SDS-PAGE (**up** panel) and Western blot (**down** panel). Lanes, MP: molecular weight marker, I: initial sample, NU: unbound, L1: washed 25 mM imidazole, L2: elution fraction with 250 mM imidazole, E: elution 500 mM imidazole, C: negative control. (**B**) Expression of pIL-18 in *P. pastoris*. SDS-PAGE (**up** panel) and Western blot (**down** panel). Lanes, C: control (epIL-18), MP: molecular weight marker, numbers represent kDa, ppIL-18: pIL-18 expressed in the culture medium of *P. pastoris*.

Porcine recombinant IL-18 was expressed through discontinuous culture using the strain *E. coli* BL21 codon plus and purified by IMAC with column renaturation. All fractions were collected: initial sample (I), unbound (NU), washes (L1 and L2), and elution (E) and analyzed by SDS-PAGE and Western blot. The epIL-18 was obtained with an estimated purity of 80% and a loss of 0.3% in the unbound fraction. However, epIL-18 was lost in the renaturation and purification process, recovering only 2% of the initial sample (Figure 2A). Cell disruption proteins of bacteria transformed with the empty vector were used as a negative control.

*P. pastoris* IL-18 expression induction was performed with a final concentration of 1% methanol for 72 h. The expression was analyzed by SDS-PAGE and Western blot, identifying an immunoreactive signal of approximately 18 kDa in the culture medium that matches the expected for the recombinant pIL-18 (Figure 2B). pIL-18 obtained from *E. coli* was used as a positive control.

### 3.3. Evaluation of the Biological Activity of IL-18

**Figure 3:**
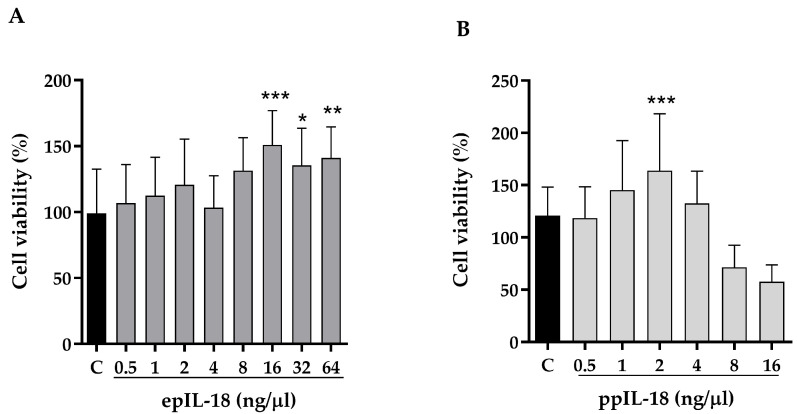
Lymphocyte proliferation assay. Lymphocytes isolated from peripheral blood were incubated with increasing concentrations of recombinant porcine IL-18. (**A**) Concentrations of 0.5, 1, 2, 3, 4, 8, 16, 32 and 64 ng/μL of epIL-18 and (**B**) 0.5, 1, 2, 3, 4, 8 and 16 ng/μL of ppIL-18, for 24 h. Cell viability was analyzed with the MTT assay to establish the proliferation of treated lymphocytes concerning the control (C). Values are mean ± SD, *n =* 3, statistical analysis was performed with one-way ANOVA, * *p* < 0.05, ** *p* < 0.01, *** *p* < 0.001.

Lymphocytes treated with pIL-18 obtained from *E. coli* (epIL-18) significantly increased their viability when incubated with 16, 32, and 64 ng/µL versus the control without treatment (Figure 3A). Moreover, lymphocytes treated with pIL-18 obtained from *P. pastoris* (ppIL-18) increased their viability significantly when incubated with 1, 2, and 4 ng/µL, respectively, compared with the control of non-transformed *P. pastoris* culture medium (Figure 3B). Statistical analysis was performed using one-way ANOVA, comparing all columns with the control group.

**Figure 4:**
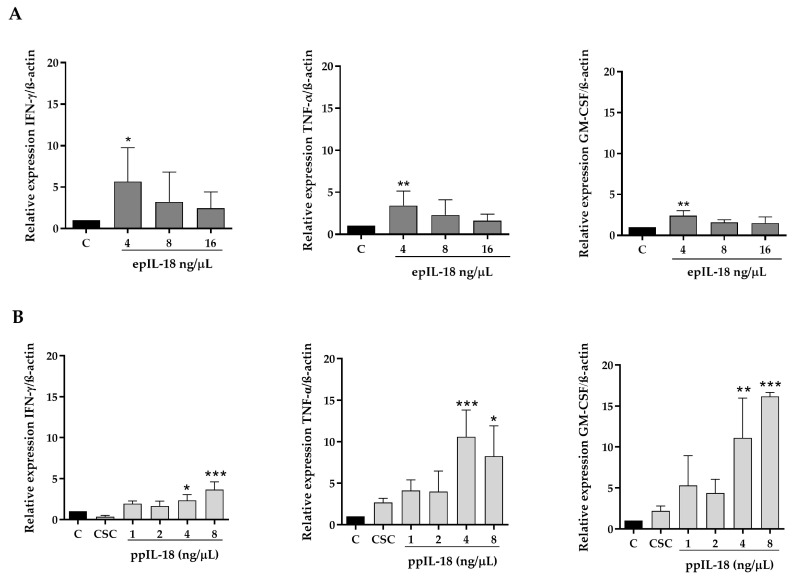
Analysis of pro-inflammatory markers in cell culture. Lymphocytes isolated from peripheral blood were incubated with (**A**) increasing concentrations of epIL-18 (1, 2, 4 y 8 ng/μL) or (**B**) ppIL-18 (4, 8 y 16 ng/μL) for 24 h before the RNA was extracted and qRT-PCR performed. The mRNA levels of the pro-inflammatory proteins IFN-γ, TNF-α, and GM-CSF increase significantly concerning the control in both proteins (epIL-18 and ppIL-18). C: control without treatment, CSC: control of culture medium of *P. pastoris* untransformed. Values are mean ± SD, *n* = 3, one-way ANOVA statistical analysis, * *p* < 0.05 ** *p* < 0.01, *** *p* < 0.001.

Peripheral blood lymphocytes were isolated and treated for 24 h with different concentrations of ppIL-18 (1, 2, 4, and 8 ng/µL) or epIL-18 (4, 8, and 16 ng/µL). Total RNA was isolated from the cells, and the relative expression of IFN-γ, TNF-α, and GM-CSF was analyzed by qRT-PCR using β-actin as the endogenous control (Figure 4).

After the treatment of PBMCs, IFN-γ relative expression in cells treated with ppIL-18 increased significantly between 4 and 8 ng/µL (Figure 4B). In lymphocytes treated with epIL-18, the relative amount of this marker increased when incubated with 4 ng/µL (Figure 4A). All data were compared with the control without treatment. Relative expression of TNF-α increased significantly in lymphocytes treated with 4 and 8 ng/µL of ppIL-18 to the control (Figure 4B). On the other hand, cells incubated with 4 ng/µL of epIL-18 increased the relative amount of this cytokine (Figure 4A). Finally, GM-CSF expression increased significantly in cells treated with 4 and 8 ng/µL of ppIL-18 concerning the control (Figure 4B). In the eplL-18 case, this marker increased in cells treated with 4 ng/µL (Figure 4A). Cells treated with the non-transformed yeast culture medium (CSC) showed no significant difference compared with the control for any of the cytokines (Figure 4B).

### 3.4. Analysis of Immunostimulation of IL-18 in Pigs

**Figure 5:**
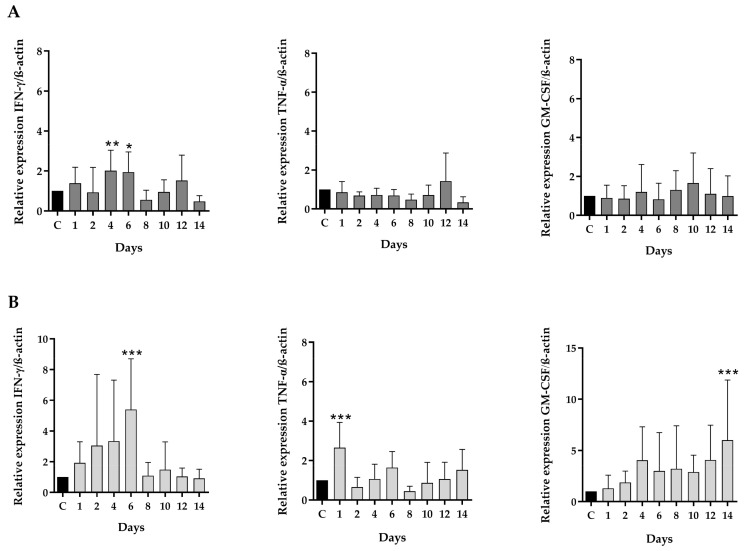
Analysis of pro-inflammatory markers in pigs. The pigs were inoculated via intramuscular injection with a formulation including (**A**) 100 μg of epIL-18 or (**B**) 100 μg of ppIL-18. Blood samples were taken, peripheral blood lymphocytes were isolated, RNA was extracted, and qRT-PCR was performed. The percentage of the mRNA levels of the genes of the pro-inflammatory proteins IFN-γ, TNF-α, and GM-CSF was compared with the control of day 0. C: day cero. Values are mean ± SD, *n =* 5, one-way ANOVA statistical analysis, * *p* < 0.05, ** *p* < 0.01, *** *p* < 0.001.

We compared both molecules’ activity in vivo, analyzing immunostimulation in pigs. IFN-γ inflammatory marker increased on day 6 post-injection concerning the control in pigs treated with the formulation based on ppIL-18 (Figure 5B). This marker increased in pigs treated with the epIL-18-based formulation on days 4 and 6 post-injection (Figure 5A). TNF-α only increased in pigs treated with ppIL-18 on day 1 post-injection. GM-CSF only increased significantly in pigs treated with ppIL-18 on day 14 post-injection. However, in the previous days, there was an upward trend in the relative amount of mRNA for this last marker (Figure 5B). Pigs treated with the epIL-18-based formulation showed no significant increase in GM-CSF (Figure 5A).

The relative expression of each cytokine in pigs treated with ppIL-18 or epIL-18 was compared. A significant difference was identified in the IFN-γ stimulation on day 6, where ppIL-18 formulation was the one that significantly stimulated the expression of this gene with a *p* < 0.001. A significant difference was also observed for the other pro-inflammatory cytokines. These results prove that the formulation based on ppIL-18 has an immunostimulatory effect more that is more significant than that based on epIL-18 (Figure 5).

### 3.5. Analysis of ppIL-18 as an Adjuvant in a Recombinant Vaccine against L. intracellularis

**Figure 6:**
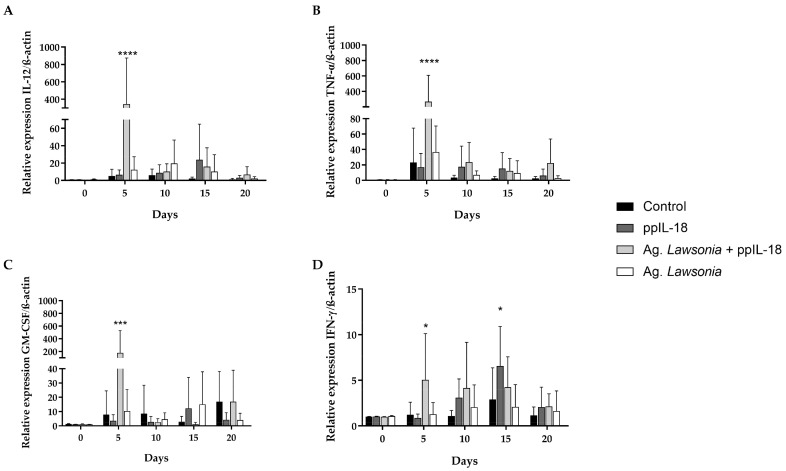
Analysis of cellular immune response in weaning pigs vaccinated with different formulations. Pigs were injected with 25 µg of ppIL-18 formulation with or without the *L. intracellularis* antigens, only the *L. intracellularis* antigens, and PBS (control). Peripheral blood lymphocytes were isolated, RNA was extracted, and qRT-PCR was performed. The relative amounts of mRNA of the cytokine genes (**A**) IL-12, (**B**) TNF-α, (**C**) GM-CSF and (**D**) IFN-γ were compared each day with the control group of each day (PBS). Values are mean ± SD, *n =* 8, two-way ANOVA statistical analysis, * *p* < 0.05, *** *p* < 0.001, **** *p* < 0.0001.

Pigs were injected with different formulations to test the ppIL-18 adjuvant effect on the subunit vaccine against *L. intracellularis*. Peripheral blood samples were taken on days 0, 5, 10, 15, and 20, and RNA was isolated and analyzed by qRT-PCR. IL-12, TNF-α, IFN-γ, and GM-CSF mRNA expression were measured. The relative expression of all these Th1 cytokines increased significantly at day 5 in pigs vaccinated with *L. intracellularis* antigens plus ppIL-18 (Ag. *Lawsonia* + ppIL-18) compared with the other 3 groups (control, ppIL-18 and Ag. *Lawsonia*) (Figure 6).

**Figure 7:**
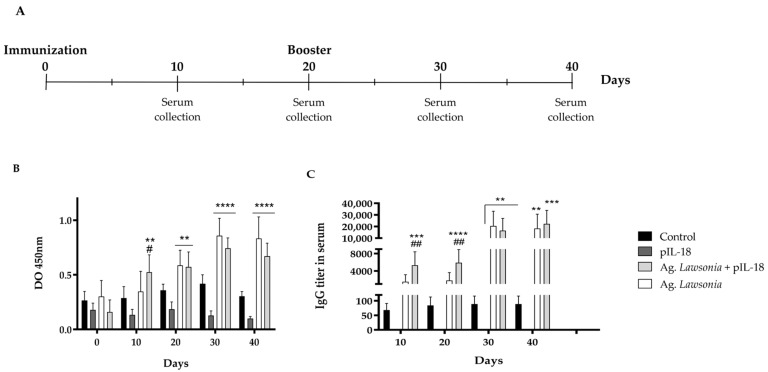
Antibody response to vaccination in pigs. Three-week-old pigs were immunized against *L. intracellularis* with or without ppIL-18, and ELISA was performed to measured IgG production. (**A**) Immunization scheme, (**B**) time course of serum IgG response to vaccination, and (**C**) IgG titer quantification in serum. Two-way ANOVA statistical analysis, ** *p* < 0.01, *** *p* < 0.001, **** *p* < 0.0001, ## *p* < 0.01 (regarding control group, # regarding Ag. *Lawsonia*).

After injection, serum samples were taken on days 10, 20, 30 and 40 to measure specific IgG antibody titer production against *L. intracellularis* (Figure 7A) by ELISA. The results show that both antigen formulations increase or stimulate the production of antibodies against *L. intracellularis* (Figure 7). However, the formulation containing the porcine ppIL-18 stimulates the IgG production 10 days before the formulation that does not contain it (Figure 7B). The produced antibodies quantification showed that on days 10 and 20, IgG titers were significantly higher in pigs injected with the antigens plus ppIL-18 than those injected with the antigens without the cytokine (Figure 7C). The maximum titer detected was 1/20,000. The vaccination with ppIL-18 by itself did not stimulate the antibody response (Figure 7B).

## 4. Discussion

In this study, we expressed the porcine IL-18 in two heterologous systems: *Escherichia coli* and *Pichia pastoris*. We compared their biological activities and selected the best of both expression systems to produce the pIL-18 to use as a vaccine adjuvant. The *E. coli* expression system is used mainly to produce recombinant biopharmaceuticals and vaccine antigens [43,44]. This system allows for the expression of heterologous proteins in a simplified system optimized for emerging needs, such as improving expression vectors and standardizing culture conditions [45]. The *P. pastoris* expression system is also used to produce recombinant proteins and biopharmaceuticals applicable to the veterinary sector. This organism, unlike *E. coli*, allows the stable integration of genetic material into its genome, generating genetically transformed clones [46,47,48,49]. *P. pastoris* is an expression system highly occupied by the ease of cultivation. This system has benefits, such as the low cost associated with its production and the high amount of recombinant proteins synthesized under the enzyme alcohol oxidase 1 promoter control (pAOX1), reaching levels of 22 g/L of intracellular proteins and 15 g/L of extracellular proteins [50,51].

The first time recombinant porcine IL-18 was cloned and expressed in *E. coli* was in 2000 [52]. In our work, the pIL-18 was expressed as inclusion bodies representing 39% of the total proteins of the bacteria, which is within the expected range reported for the expression of heterologous proteins in this system [43,53]. We used the technique of re-folding in packed chromatographic columns to regenerate the protein [54,55]. On the other hand, the pIL-18 produced in *P. pastoris* was not purified by IMAC because it did not bind the matrix. We evaluated the histidine tag exposure by homology modelling and observed that 6His-tag was not completely exposed (Figure S1), which can explain why it did not bind to the purification column. However, as *P. pastoris* is classified as a GRAS microorganism [46,56], it was possible to use pIL-18 without purifying it from the culture medium. It should be noted that using purification techniques such as affinity chromatography increases the production cost of biopharmaceuticals. This fact is an essential factor to consider in the veterinary industry, where it is expected to have low-cost products that do not affect the prices of breeding and fattening pigs.

The epIL-18 and ppIL-18 biological activity was evaluated in pig peripheral blood lymphocyte cultures using the MTT colorimetric assay [57,58]. The increased protein concentrations were lower than those reported in porcine IL-18 expressed in *E. coli* [57]. Moreover, ppIL-18 showed the same biological effect as epIL-18, but at 16 times lower concentration, indicating that it has a more significant impact than epIL-18 (Figure 3). This difference in both variants’ activity can be attributed to the differences in the expression conditions. The in vitro immunostimulation results show that both variants of pIL-18 induce the expression of the three immune markers at the same concentration (Figure 4). Previous studies have shown that human IL-18 increases TNF-α at 12 h in PBMCs treated with IL-18 at 10 nM and increased IFN-γ at the same concentration at 24 h [59,60]. In this work, we detected the markers induction at 4 ng/μL of IL-18, equivalent to 222 nM. IFN-γ is induced in the same proportion by epIL-18 and ppIL-18 without significant differences between them. However, the TNF-α and GM-CSF induction with 4 ng/μL had a significant difference between the molecule produced in *E. coli*, increasing approximately 5 times the relative expression of both molecules, compared with the molecule produced in *P. pastoris*, which increases the relative expressions of those markers in over 10 times; these results prove that ppIL-18 has a greater effect on these markers than epIL-18 (Figure 4). We can suggest that the difference in the immunostimulatory effects observed for both variants of pIL-18 is because of an optimal structural conformation of the secreted molecule in *P. pastoris*, compared with the possible affectations that can be structurally generated in the variant expressed in *E. coli*. Developing chemical denaturation and renaturation steps is necessary to obtain the soluble molecule. On the other hand, the protein expressed in *E. coli* was not obtained with 100% purity, and the endotoxin presence was not analyzed, so we are not sure that these impurities are not causing damage to the cells or interfering with the IL-18 biological activity.

The in vivo studies in adult pigs showed that ppIL-18 induced all three markers’ expression. On the contrary, epIL-18 seems to stimulate only the expression of IFN-γ, which indicates that ppIL-18 had a more significant immunostimulatory effect in adult pigs than its counterpart epIL-18, consistent with the results obtained in the in vitro analysis.

Our results are supported by other publications showing the induction markers by IL-18 in response to infection. Neighbors et al. have demonstrated that IL-18 induces TNF-α and nitric oxide production in response to infection with *Listeria monocytogenes* [61]. A study of the reaction in infection with *Mycobacterium tuberculosis* postulates that IFN-γ increases the expression of TNF-α and the IL-18 receptor in uninfected macrophages and the presence of the infectious agent at 4 h of treatment. In turn, TNF-α increases the IFN- γ receptor expression at 4 h of treatment in the presence or absence of the infectious agent [62]. In addition, the importance of the IL-18 response in the infection against the dengue virus has been demonstrated, dependent on IL-18 and the production of IFN-γ [63]. Additionally, IL-18 stimulates CD4+ cells and macrophages to secrete GM-CSF and other cytokines. They induce hematopoietic cells and proliferation-causing neutrophilia and eosinophilia in mice [64]. This induction is independent of IFN-γ [64].

The difference in times when the marker increments were detected can be justified by integrating the previous information, which indicates that the IFN-γ/TNF-α/GM-CSF responses may or may not be interfered with by themselves. For example, IFN-γ could induce the GM-CSF expression in lymphocytes, explaining the increase in this marker several days after the maximum IFN-γ expression. On the other hand, the organism’s immune response always depends on the context in which it finds itself. Therefore, the lack of TNF-α stimulation can be associated with the infectious agent absence in the test or because GM-CSF does not present an early induction (it was expressed on day 14). The TNF-α expression stimulation could occur at a time after the study range.

To assess the use of our IL-18 expressed in *P. pastoris* as a vaccine adjuvant, a vaccine candidate against *L. intracellularis* was used [24]. Pigs vaccinated with the candidate co-formulated with ppIL-18 showed a significant immune marker expression peak of the Th1 response (IL-12, TNF-α, IFN-γ, and GMCSF) at day 5 post-administration, compared with the control groups and with the group with the vaccine alone (Figure 6). Although these data coincide with the increase in IL-12 reported on day 4 post-administration [24], the increase in relative expression with IL-18 is significantly higher. In vitro studies, where pig’s peripheral blood lymphocyte cultures were naturally infected with *Lawsonia intracellularis*, when stimulated with IL-18, showed IFN-γ increases at 24 h post-stimulation [65]. In addition, in DNA vaccines in pigs, an increase in this marker in the serum of animals when using IL-18 in conjunction with the antigen of interest, has also been observed [66,67].

Previously, Montesino et al. have reported a significant increase in antibodies at week 4 post-administration of the *L. intracelullaris* vaccine candidate [24]. However, when quantifying the antibody titers, we can observe significant differences from day 10 in the pigs injected with the vaccine candidate co-formulated with ppIL-18, which is equal to the group with the vaccine candidate at day 30 post-administration (Figure 7). Antigen co-formulation with ppIL-18 increases the antibody titer by 20 days compared with the group vaccinated only with the antigens. This result indicates that the use of IL-18 as a vaccine adjuvant with these recombinant antigen formulations induces a positive effect in a shorter period, even before the booster, with a reduction in the therapeutic window under this vaccination strategy.

The co-formulation vaccine candidate with ppIL-18 improves the cellular immune response by increasing the Th1 response and the humoral immune response by producing specific IgG at a systemic level. Th1 response is vital for invading microorganisms such as *L. intracellularis*, stimulating the CD4+ and CD8+ cell response to eliminate infected cells and produce specific cytokines [68].

Previously, the immunostimulatory effect of porcine IL-18 was assessed in mice and pigs using *Erysipelothrix rhusiopathiae* expressing IL-18, inducing cellular and humoral immune responses against different antigens [69]. On the other hand, the vaccine adjuvant IL-18 has been proven, for example, in a DNA vaccine against Newcastle disease [70,71,72]. Another DNA vaccine was administered in PLGA microparticles against foot-and-mouth disease [73]. IL-18 has been used as an adjuvant in recombinant bacterial vaccines against porcine circovirus type 2 in pigs [74] and coccidiosis in chickens [75]. Additionally, IL-18 was evaluated in a universal influenza virus vaccine, with the most promising results compared with the other cytokines assessed in that study [76]. These studies support the idea of IL-18 as an immunostimulant and vaccine adjuvant to enhance the immune response against the desired antigen.

Mucosal immune response, specifically IgA antibodies are also relevant for this microorganism, which invades the mucous membranes, specifically the digestive system [77,78]. Evaluating the stimulation of these immune responses in pigs is necessary to cover the entire immune response essential for this pathogen control.

## 5. Conclusions

IL-18 produced in *P. pastoris* showed increased biological activity both in vitro and in vivo. Additionally, its use as an adjuvant in the vaccine candidate against *L. intracellularis* elicits an earlier humoral immune response than the vaccine itself, reducing the therapeutic window. This work demonstrates and confirms the potential use of recombinant porcine IL-18 as an enhancer of the immune response in vaccines, specifically in a vaccine candidate based on recombinant proteins.

## Data Availability

All relevant data are presented in the article.

## Supplementary Materials

The following supporting information can be downloaded at: https://www.mdpi.com/article/10.3390/vaccines11121788/s1, Table S1: Results obtained from the search for the most suitable templates for constructing the models by homology for IL-18, Figure S1: Homology modelling of the histidine tail of IL-18.
